# Factors associated with recent HIV testing among high-risk men who have sex with men: a cross-sectional study in Cambodia

**DOI:** 10.1186/s12889-015-2096-4

**Published:** 2015-08-01

**Authors:** Siyan Yi, Sovannary Tuot, Pheak Chhoun, Carinne Brody, Khuondyla Pal, Sopheap Oum

**Affiliations:** Research Center, KHANA, No. 33, Street 71, P.O Box 2311-PP3, Phnom Penh, Cambodia; Center for Global Health Research, Public Health Program, Touro University California, 1310 Club Drive, Vallejo, CA 94594 USA

**Keywords:** Men who have sex with men, HIV testing, Risky sexual behaviors, Condom use, Cross-sectional study, Cambodia

## Abstract

**Background:**

Despite remarkable success in the fight against HIV, HIV prevalence in many countries remains high among key populations including men who have sex with men (MSM), and HIV testing rates is relatively low among this hard-to-reach population. This cross-sectional study explores factors associated with recent HIV testing among MSM in Cambodia.

**Methods:**

This study was conducted in 2014 and included 384 MSM randomly selected from two provinces of Battembang and Siem Reap, using a two-stage cluster sampling method. A structured questionnaire was used for face-to-face interviews to collect data on socio-demographic characteristics, HIV testing history, sexual behaviors, HIV testing attitudes, and HIV knowledge. Multivariate logistic regression analysis was performed to identify factors independently associated with recent HIV testing.

**Results:**

Mean age of the participants was 23.4 (SD = 5.2). Of total, 83.6 % had been tested for HIV at least once in their lifetime, and 65.1 % had been tested for HIV in the past six months. After controlling for other covariates, MSM who had been tested for HIV in the past six months were significantly more likely to regard themselves as female (AOR = 2.29, 95 % CI = 1.06-5.37), have received some form of HIV education in the past six months (AOR = 3.97, 95 % CI = 1.91-8.26), perceive that they were at higher HIV risk compared to the general population (AOR = 2.48, 95 % CI = 1.14-4.86), have been diagnosed with an STI in the past six months (AOR = 3.19, 95 % CI = 1.02-9.24), report using a condom at last sexual intercourse with a man or woman (AOR = 2.24, 95 % CI = 1.06-3.13), and report using a condom at last sexual intercourse with a boyfriend (AOR = 2.17, 95 % CI = 1.04-5.31).

**Conclusions:**

This study highlights the common practices of risky sexual behaviors and relatively low rate of recent HIV testing among MSM in Cambodia. HIV education and social marketing should be expanded and tailored for MSM, specifically addressing the risk of unprotected anal intercourse and the importance of regular HIV testing for early enrolment in the care and treatment cascade.

## Background

Cambodia has been recognized for successful HIV prevention and control efforts that have contributed to a decline in HIV prevalence in the general population aged 15 to 49 from approximately 2 % in late 1990’s to 0.6 % in 2013 [1, 2]. The country has also achieved the universal access target for antiretroviral therapy (ART), with over 90 % of people living with HIV (PLHIV) in need for ART receiving the treatment [1]. Moreover, annual AIDS-related deaths have been reduced by two-thirds over the past 10 years [2]. As a result, Cambodia received a Millennium Development Goal award from the United Nations in 2010 [3]. Despite these remarkable achievements, Cambodia is facing emerging challenges in the concentrated HIV epidemic in key populations. HIV prevalence remains very high among female entertainment workers (FEWs), injecting drug users (IDUs), men who have sex with men (MSM), and transgender people (TG) [2, 4, 5].

The concerns about resurgence of HIV infection among MSM first started in the Western world [6–8], and emerged later on in Latin America, Africa, and Asia [9–12]. The substantial risk for HIV infection among MSM has been reported in different socio-cultural contexts throughout the world [13–17]. Because of the hidden and stigmatized nature of MSM, there are data gaps and challenges to research, surveillance, and epidemiological characterization in MSM populations [18]. HIV prevention and care among these populations remains challenging. Recent studies have consistently reported that HIV testing rates remain low among MSM in many countries [19–26]. In Cambodia, the BROS Khmer Study conducted in 2010 found that 60 % of the study sample reported having been tested for HIV in their lifetime, and among these men, 64 % reported having had an HIV test in the past 12 months [5]. According to the most recent Behavioral Sentinel Surveillance (BSS 2013), approximately 87 % of MSM had been tested for HIV in the past 12 months, and 70 % had been tested for HIV in the past three months [27]. Among MSM in the BROS Khmer Study, the prevalence of HIV and sexually transmitted infections (STIs) were 2.2 % and 52 %, respectively [5].

Regular HIV testing, particularly in key populations with high HIV prevalence, plays a very important role in reducing new HIV infections through early HIV detection, and it is the first step in engaging infected people in the care and treatment cascade [24]. Early detection not only reduces the chance of HIV transmission but also enables timely treatment to reduce HIV-related mortality and morbidity [28]. Moreover, a recent Cochrane review of studies in many developing countries found that HIV voluntary confidential counseling and testing (VCCT) is effective in increasing case detection and reducing risky sexual behaviors [29]. Promoting HIV testing is thus very important in HIV prevention, care, and support for at-risk MSM. The World Health Organization (WHO) recommends that MSM with high-risk behaviors should receive HIV testing every six to 12 months [30], while the U.S Center for Disease Control and Prevention (CDC) recommends sexually active MSM to get tested for HIV every three to six months [31]. These goals are reached only if barriers to HIV testing are identified, and effective strategies to overcome these barriers are put in place.

Several factors associated with HIV testing among MSM have been identified in different settings. These factors include characteristics such as age, education, mobility, sexual behaviors, perception of HIV risk, perceived stigma and discrimination towards MSM in communities and health facilities, HIV testing attitudes, perceived necessity of HIV test, and fears of positive results [20–22, 24–26, 32, 33]. However, several findings in these existing studies were not consistent. For examples, studies among MSM in Chongqing, China [22, 26] found that HIV testing was associated with the report of protected sexual intercourse, while another study in Beijing, China found that HIV testing was associated with the report of unprotected sexual intercourse [33]. Similarly, the relationship between HIV testing and perceived low HIV risk was found in another study in mainland China [26] and Thailand [25], while a study in Hong Kong found that HIV testing was associated with greater perception of chance of having sexual intercourse with HIV-infected people in the next six months [21]. Other studies also reported that perception of low HIV risk was also one of the reasons for not having been tested for HIV [23, 34].

The discrepancy in the findings in different populations may indicate the effects of socio-cultural aspects of MSM on the relationships between HIV testing and related factors and merits further investigations. Moreover, most of the available studies in the literature have examined factors associated with history of lifetime HIV testing [24–26, 34], HIV testing in the past 12 months [35, 36], or intention to take up HIV testing [20, 21]. Findings from these studies are definitely important; however, it is more critical to understand factors related to more recent HIV testing history. The Cambodia’s national guideline recommends HIV testing at least once in six months for sexually active MSM to identify new HIV infections and prevent ongoing transmission [1]. To address this literature gap, we conducted this cross-sectional study to explore factors associated with recent HIV testing (in the past six months) among MSM in Cambodia.

## Methods

### Study sites and population

Data were collected in April and May 2014 as part of an impact evaluation of the Sustainable Action against HIV and AIDS in Communities (SAHACOM) project. The details of the main study have been published elsewhere [37–40].

In brief, data were collected from 394 MSM randomly selected from venues and hotspots identified by community-based workers in two provinces of Battembang and Siem Reap provinces. A two-stage cluster sampling method was used to select the study sample with communes in each province as the smallest unit for the sampling. We included only communes with at least 20 MSM; and other conditions, such as convenience and accessibility, were also considered to justify whether to include or exclude a venue or hotspot. We then used the probability proportional-to-size sampling to select the required number of MSM from each commune. Inclusion criteria for participation in this study were: (1) age of 18 years or older; (2) self-reported as an MSM; (3) having had sexual intercourse experience; (4) being able to provide consent to participate in the study; and (5) being able to present themselves on the day of the data collection. In data cleaning, we found ten respondents (2.5 %) who did not have sexual experience, and they were excluded from the analyses.

### Questionnaire development and training

A structured questionnaire was initially developed in English and translated into Khmer, the national language of Cambodia, and it was then back-translated into English by another translator. A pilot study was conducted with a sample of 10 MSM in Phnom Penh to ensure that the wording and contents were culturally suitable, acceptable, and clearly understandable for the participants. Necessary modifications were made after receiving comments from experts working on HIV in key populations in Cambodia as well as feedbacks from the pilot study.

A three-day training was conducted for all research team members on data collection methods and tool pretesting and reflection. The contents of training included privacy assurance, confidentiality, interview techniques, and reviews of the study protocol and questionnaire. Research team members were also equipped with quality control skills such as rechecking and reviewing the questionnaires after administration as well as resolving issues that might arise during the fieldwork. We encouraged data collection team leaders to perform regular review sessions with interviewers to review progress and communicate any issues occurring during the data collection.

### Variables and measurements

We adapted standardized tools from a previous study in the same population [41], the most recent Cambodia Demographic and Health Survey [42], as well as from other studies in Cambodia [43, 44] to measure socio-economic characteristics, perceived HIV risk, sexual behaviors, history of STIs, history of HIV testing, health care seeking behaviors, and HIV education.

For socioeconomic characteristics, we collected information on age, marital status, years of formal education completed, main occupation, average monthly income, living situations, and personal perception about sexual identity. To assess history of HIV testing, a yes/no question was used, “In the past six months, have you been tested for HIV?” Condom use was measured by using yes/no questions asking whether they used a condom at last sexual intercourse with different types of partners such as regular female partners, regular male partners, female sex workers, male sex workers, female clients, and male clients. Yes/no questions were also used to collect information on several other sexual behaviors in the past three months with the above-mentioned partners. We also collected data on HIV education they received in the past six months, STI symptoms in the past three months, and illicit drug use in the past six months.

HIV testing attitudes were measured using five items adapted from a previous study on HIV testing and testing attitudes [45]. Two items reflected positive outcomes from testing, two assessed adverse outcomes, and one item reflected HIV testing avoidance. The participants responded to each statement dichotomously, as either “agree” or “disagree.” To measure HIV knowledge, we used a 12-item scale that reflected information about HIV transmission, condom use, and HIV knowledge [46]. The items were answered as ‘yes,’ ‘no,’ or ‘don’t know’ with ‘don’t know’ responses regarded as incorrect.

### Ethical considerations

This study was approved by the National Ethics Committee for Health Research, Ministry of Health, Cambodia (Ref no. 082NECHR). Participants were made clear that participation in this study was voluntary, and they could refuse or discontinue their participation at any time for any reason. We obtained a written informed consent from each participant after a detailed description of the study was provided. To ensure the confidentiality, no personal identifiers were collected, and code numbers were used. Privacy of the respondents was protected by conducting the interviews at a private place.

### Data analyses

EpiData version 3 (Odense, Denmark) was used for double data entry, and SPSS version 22 (IBM Corporation, New York, USA) was used for all statistical analyses. Chi-square test or Fisher’s exact test was used as appropriate for categorical variables, and Student’s *t*-test was used for continuous variables to compare socio-demographic characteristics, sexual behaviors, HIV testing attitudes, and HIV knowledge among MSM who had and who had not been tested for HIV in the past six months. A multivariate logistic regression analysis was performed to examine factors independently associated with recent HIV testing, controlling for potential confounders. Variables significantly associated with HIV testing in bivariate analyses at a level of *p* < 0.2 were simultaneously included in the model. In the model, variables with a *p*-value >0.05 were then removed for model fitting, and the steps were repeated until all *p*-values of the remaining variables were <0.05 in the final model. Multicollinearity was detected between condom use and lubricant use in last anal intercourse with a boyfriend; the variable for lubricant use was thus excluded from the model. Adjusted odds ratio (AOR) were calculated and presented with 95 % confidence intervals (CI) and *p*-values.

## Results

### History of HIV testing

This study included 384 MSM with a mean age of 23.4 (SD = 5.2). On average, 83.6 % of the participants reported having been tested for HIV at least once in their lifetime, and 65.1 % had been tested in the past six months. They received their most recent HIV testing at a VCCT site (45.9 %), through community- or peer-initiated HIV testing (43.3 %), or at a private facility (10.8 %). Most of them (65.2 %) were referred by peer educators, outreach workers, or other NGO staff for HIV testing; 19.9 % were self-referred; and 10.2 % were referred by friends or colleagues. The majority received the result (98.2 %) and counselling (95.7 %) for the most recent testing. When asked about the reasons for not getting tested, more than half (56.3 %) of participants who had not been tested for HIV in the past six months responded that they did not think they were at risk for HIV, and 15.0 % raised their fear of positive result as the main reason for not getting tested.

### Comparisons of socio-demographic characteristics

Table 1 shows comparisons of socio-demographic characteristics of MSM who had and who had not been tested for HIV in the past six months. More than half (56.8 %) of the respondents regarded themselves as male, and 21.1 % and 22.1 % regarded themselves as female and both genders, respectively. The majority (90.1 %) were never married with mean years of formal education completed of 9.5 (SD = 3.2). The most common job was self-employed (29.6 %), followed by students (26.6 %) and other jobs such as working as a hairdresser, a barber, or in a beauty salon shop (23.4 %). Their average monthly income was $205 (SD = $613). More than two-thirds (71.4 %) lived with their parents, and 84.9 % had received some form of HIV education in the past six months. When asked to compare level of their HIV risk, 35.4 % perceived themselves as having higher risk; 18.0 % perceived themselves as having the same risk, and 46.6 % perceived themselves as having lower risk compared to the general population.

**Table 1:** Comparisons of socio-demographic characteristics of MSM who had and who had not been tested for HIV

Socio-economic characteristics		Total (*n* = 384)	HIV testing in the past 6 months		
			No (*n* = 134)	Yes (*n* = 250)	*p*-value^*^
Mean age (in year)		23.4 ± 5.2	23.4 ± 4.4	24.1 ± 5.6	0.18
Perceived sexual identity					<0.001
	Male	218 (56.8)	95 (70.9)	123 (49.2)	
	Female	81 (21.1)	13 (9.7)	68 (27.2)	
	Both	85 (22.1)	26 (19.4)	59 (23.6)	
Marital Status					0.70
	Never married	345 (90.1)	120 (89.6)	225 (90.4)	
	Married	30 (7.8)	12 (9.0)	18 (7.2)	
	Divorced, separated, or widowed	8 (2.1)	2 (1.5)	6 (2.4)	
Years of formal education completed		9.5 ± 3.2	9.4 ± 3.5	9.6 ± 3.1	0.53
Main occupations					0.24
	Unemployed	16 (4.3)	7 (5.3)	9 (3.8)	
	Students	98 (26.6)	38 (28.8)	60 (25.4)	
	Farmer/laborer	59 (16.0)	24 (18.2)	35 (14.8)	
	Self-employed	109 (29.6)	41 (31.1)	68 (28.8)	
	Other	86 (23.4)	22 (16.7)	64 (27.1)	
Mean monthly income (in US$)		205 ± 613	211 ± 738	201 ± 536	0.89
Currently living with:					0.007
	Parents	269 (71.4)	99 (75.6)	170 (69.1)	
	Relatives/siblings	37 (9.8)	3 (2.3)	34 (13.8)	
	Spouse/sexual partner	32 (8.5)	15 (11.5)	17 (6.9)	
	Friends/colleagues	25 (6.6)	9 (6.9)	16 (6.5)	
	Other	14 (3.7)	5 (3.8)	9 (3.7)	
Mean years living in current city		19.6 ± 8.8	18.7 ± 9.0	20.0 ± 8.6	0.15
Received HIV education (past 6 months)		325 (84.9)	96 (72.2)	229 (91.6)	<0.001
Sources of HIV education received in the past 6 months					
	Media (TV/radio/newspaper)	188 (57.7)	64 (66.0)	124 (54.1)	0.04
	Poster/billboard/booklet	85 (26.1)	25 (25.8)	60 (26.2)	0.94
	Peer educator/outreach workers	295 (90.5)	85 (87.6)	210 (91.7)	0.25
	Counseling at VCCT	16 (4.9)	5 (5.2)	11 (4.8)	0.89
	Public health facilities	33 (10.1)	7 (7.2)	26 (11.4)	0.26
	Other	33 (10.1)	10 (10.3)	23 (10.0)	0.94
Perception of HIV risk compared to the general population					0.007
	Higher	136 (35.4)	34 (25.4)	102 (40.8)	
	Same	69 (18.0)	31 (23.1)	38 (15.2)	
	Lower	179 (46.6)	69 (51.5)	110 (44.0)	

*MSM* men who have sex with men, *TV* television, *VCCT* voluntary confidential counseling and testing

Values are number (%) for categorical variables and mean ± SD for continuous variables

^*^Chi-square test or Fisher’s exact test was used for categorical variables and Student’s *t*-test was used for continuous variables

Compared to participants who had not been tested for HIV in the past six months, those who had been tested were significantly more likely to regard themselves as female (27.2 % vs. 9.7 %, *p* < 0.001) and less likely to live with their parents (69.1 % vs. 75.6 %, *p* = 0.007). They were also significantly more likely to have received some form of HIV education in the past six months (91.6 % vs. 72.2 %, *p* < 0.001) and to perceive themselves as having higher HIV risk compared to the general population (40.8 % vs. 25.4 %, *p* = 0.007).

### Comparisons of sexual behaviors

Comparisons of sexual behaviors among participants who had and who had not been tested for HIV in the past six months are shown in Table 2. Mean number of sex partners in the past three months was significantly higher among participants who had been tested for HIV (3.2 ± 3.8 vs. 4.5 ± 6.2, *p* = 0.02). Participants who had been tested were significantly more likely to report using a condom at last sexual intercourse with a man or woman (87.4 % vs. 74.2 %, *p* = 0.001) and at last sexual intercourse with a girlfriend (89.0 % vs. 71.1 %, *p* = 0.01). They were significantly less likely to have a girlfriend (44.4 % vs. 60.4 %, *p* = 0.003), but more likely to have a boyfriend (69.2 % vs. 49.3 %, *p* < 0.001) in the past three months. Participants who had been tested for HIV were also significantly more likely to report using a condom (95.4 % vs. 82.4 %, *p* = 0.003) and lubricant (85.4 % vs. 62.0 %, *p* < 0.001) at last anal intercourse with a boyfriend, and less likely to have sexual intercourse with female sex workers in the past three months (11.6 % vs. 20.1 %, *p = 0.03*). They were also significantly more likely to have been diagnosed with an STI in the past six months (9.6 % vs. 2.3 %, *p* = 0.006).

**Table 2:** Comparisons of sexual behaviors among MSM who had and who had not been tested for HIV

Sexual behaviors in the past 3 months	Total (*n* = 384)	HIV testing in the past 6 months		
		No (*n* = 134)	Yes (*n* = 250)	*p*-value^*^
Mean number of sex partners	4.0 ± 5.5	3.2 ± 3.8	4.5 ± 6.2	0.02
Condom use at last sexual intercourse	313 (82.8)	98 (74.2)	215 (87.4)	0.001
Had a girlfriend	192 (50.0)	81 (60.4)	111 (44.4)	0.003
Had sexual intercourse with a girlfriend	118 (61.5)	45 (55.6)	73 (65.8)	0.15
Mean number of girlfriends you had sex with	1.7 ± 1.1	1.8 ± 1.3	1.7 ± 1.0	0.63
Condom use at last sex with a girlfriend	97 (82.2)	32 (71.1)	65 (89.0)	0.01
Had a boyfriend	239 (62.2)	66 (49.3)	173 (69.2)	<0.001
Had sexual intercourse with boyfriends	205 (86.9)	53 (81.5)	152 (88.9)	0.14
Mean number of boyfriends you had sex with	2.4 ± 3.9	1.8 ± 2.0	2.6 ± 4.3	0.20
Condom use in last anal sex with a boyfriend	186 (92.1)	42 (82.4)	144 (95.4)	0.003
Lubricant use in last anal sex with a boyfriend	160 (79.6)	31 (62.0)	129 (85.4)	<0.001
Had sexual intercourse with female sex workers	56 (14.6)	27 (20.1)	29 (11.6)	0.03
Condom use in last sex with female sex workers	52 (92.9)	24 (88.9)	28 (96.6)	0.34
Had sexual intercourse with male sex workers	38 (9.9)	13 (9.8)	25 (10.0)	0.94
Condom use in last sex with male sex workers	35 (94.6)	11 (91.7)	24 (96.0)	0.59
Sold sex to women	34 (8.9)	14 (10.4)	20 (8.0)	0.42
Condom use in last time selling sex to women	32 (97.0)	13 (92.9)	19 (100)	0.42
Sold sex to men	67 (17.4)	18 (13.4)	49 (19.6)	0.13
Condom use in last time selling sex to men	63 (94.0)	16 (88.9)	47 (95.9)	0.29
Always used lubricant when selling sex to men	41 (64.1)	9 (50.0)	32 (69.6)	0.14
Diagnosed with an STI in past 6 months	27 (7.1)	3 (2.3)	24 (9.6)	0.006

*MSM* men who have sex with men

Values are number (%) for categorical variables and mean ± SD for continuous variables

^*^Chi-square test or Fisher’s exact test was used for categorical variables and Student’s *t*-test was used for continuous variables

### HIV testing attitudes and knowledge

In general, MSM in this study expressed positive attitudes towards HIV testing. For example, 95.6 % agreed that getting tested for HIV helps people feel better, and only 30.7 % supported the statement that, “I would rather not know if I have HIV.” No significant difference was found in the comparisons of HIV testing attitudes among participants who had and who had not been tested for HIV in the past six months. Similarly, the majority of MSM in this study responded correctly to most of HIV knowledge questions. A large knowledge gap was noted in a question regarding if a person must have many different partners to get HIV. No significant difference was found in comparisons of HIV knowledge among participants who had and who had not been tested for HIV in the past six months.

### Factors associated with recent HIV testing

Table 3 shows factors which remained significantly associated with recent HIV testing in the multivariate logistic regression model. After controlling for other covariates, participants who had been tested for HIV in the past six months were significantly more likely to regard themselves as female (AOR = 2.29, 95 % CI = 1.06-5.37), have received some form of HIV education in the past six months (AOR = 3.97, 95 % CI = 1.91-8.26), perceive that they were at higher HIV risk compared to the general population (AOR = 2.48, 95 % CI = 1.14-4.86), have been diagnosed with an STI in the past six months (AOR = 3.19, 95 % CI = 1.02-9.24), report using a condom at last sexual intercourse with a man or woman (AOR = 2.24, 95 % CI = 1.06-3.13), and report using a condom at last anal intercourse with a boyfriend (AOR = 2.17, 95 % CI = 1.04-5.31).

**Table 3:** Factors associated with recent HIV testing among MSM in a multiple logistic regression model

Variables in the model^*^		HIV testing in the past 6 months	
		AOR (95 % CI)	*p*-value
Gender identity			
	Male	Reference	
	Female	2.29 (1.06-5.37)	0.04
	Both	1.13 (0.59-2.13)	0.71
Received some form of HIV education in the past 6 months			
	No	Reference	
	Yes	3.97 (1.91-8.26)	<0.001
Perception of HIV risk compared to the general population			
	Same	Reference	
	Higher	2.48 (1.14-4.86)	0.02
	Lower	1.16 (0.53-2.35)	0.56
Diagnosed with an STI in the past 6 months			
	No	Reference	
	Yes	3.19 (1.02-9.24)	0.04
Condom use in last sexual intercourse with a man or a woman			
	No	Reference	
	Yes	2.24 (1.06-3.13)	0.03
Condom use in last anal intercourse with a boyfriend			
	No	Reference	
	Yes	2.17 (1.04-5.31)	0.04

*AOR* adjusted odds ratio, *CI* confidence interval; *MSM* men who have sex with men

^*^Variables in the table were the ones that remained statistically significant after several steps of model fitting

## Discussion

The majority of MSM in this study had been tested for HIV at least once in their lifetime, and about two-thirds had been tested in the past six months. These rates are higher than the rates reported in 2010 [5] and similar to the results from the national BSS 2013 [27]; however, it remains far below the goal recommended by the WHO [30] and CDC [31]. In Cambodia, HIV testing and counselling services have been widely offered free of charge at VCCT centers and in communities through an extensive network of community support volunteers and peer outreach workers who work closely with key populations including MSM.

We found that MSM who had been tested for HIV in the past six months were more likely to have received some form of HIV education in the past six months. HIV education is a core component of community-based intervention programs in Cambodia that might be effective in reducing risky sexual behaviors and increasing access to HIV prevention and care services through partnership with MSM networks [2]. Collaboration between community support volunteers and peer outreach workers at MSM hotspots play a very important role in improving access to HIV education as well as HIV and STI screening for this hard-to-reach population. Future HIV prevention and care programs for MSM should emphasize the importance and advantages of knowing one’s HIV status. Our data showed that peer educators and outreach workers were the main source of HIV education for MSM, indicating that community-based approaches that have been employed in intervention programs, such as the SAHACOM project, are crucial in reaching MSM with education and focused prevention [47]. To maximize the effectiveness of the education, the contents of the materials used for the education should be regularly reviewed and updated with active involvement from community workers and stakeholders.

Risk perception seems to play a key role in MSM’s decision whether to get tested for HIV. MSM who perceived themselves at higher HIV risk compared to the general population were more likely to get tested for HIV in the past six months compared to those who thought they were at the same risk. Moreover, when asked about the reasons for not getting tested, more than half responded that they did not think they were at risk for HIV. These findings do not support previous studies in which perception of high HIV risk may prohibit MSM from getting tested [25, 26, 33, 36, 48]. However, they are in line with a study among MSM in Hong Kong, which reported that MSM who perceived higher chance of having sexual intercourse with HIV-infected people in the next six months were more likely to report having been tested for HIV [21]. This finding indicates a need for greater awareness of HIV risk among MSM in Cambodia as almost 60 % of the respondents perceived the level of their HIV risk to be the same or lower than that in the general population, despite their active involvement in risky sexual behaviors. On the other hand, it may highlight stronger supports in care and treatment services for HIV-infected people in some countries such as Cambodia and Hong Kong that may encourage high-risk MSM to get tested.

In this study, MSM who had been tested for HIV in the past six months were more likely to report using a condom at last sexual intercourse with male or female partners and at last anal intercourse with a boyfriend. A previous study in New Zealand reported that HIV testing was significantly associated with consistent condom use during anal intercourse with casual partners, but condom use with regular partners was not a factor [35]. Perception of high likelihood of having unprotected anal intercourse was associated with lower intention to take up VCCT services among MSM in Hong Kong [21]. This finding could be interpreted in another way that MSM who were involved in unprotected sex may not want to get tested because of fear of positive result. Fear of testing positive is closely related to stigma and discrimination [49, 50], and was the second most common reason for not getting tested among MSM in our study. Fear of consequences of a positive result is common among MSM who are involved in risky sexual behaviors and has been found to be the main reason for not getting tested [19, 23, 25, 26]. However, in contrast, many other studies found that MSM with more experience of risky sexual behaviors were more likely to undergo HIV testing because of their awareness of the risk involvement, and HIV testing is used to confirm or rule out the possibility of the transmission [22, 33].

HIV testing attitudes and knowledge were generally good but were not associated with recent HIV testing among MSM in this study. These findings do not necessarily indicate that HIV education is not important for MSM in Cambodia but may suggest the need for development of HIV education materials specifically designed for MSM. Previous studies have highlighted the importance of inclusion of specific knowledge items for MSM such as the information that unprotected anal intercourse exposes MSM to higher risk of HIV and STI transmission than vaginal intercourse, as well as the importance of lubricant use in anal intercourse [51, 52]. Such knowledge may change their HIV risk perception, and in turn lead to the increased uptake of HIV testing among MSM.

The major limitation of this study concerns the representativeness of the study samples as we included only MSM in two provinces where the SAHACOM, a comprehensive community-based HIV project, has been implemented for MSM. The levels of HIV risk and outcomes reported in this study may therefore represent a more optimistic view than in other areas of Cambodia. Future studies should include a wider range of MSM subpopulations; particularly those who are not reached by the intervention programs. Second, data used for this analysis were collected as part of an impact evaluation of the SAHACOM project. Thus the sampling scheme was not necessarily designed for this cross-sectional analysis. Third, the self-reported measures may lead to inherent biases potential for both underreporting and over-reporting. However, measures were taken to create conditions that encouraged valid responses from the respondents; their responses were confidential, and interviews were conducted in a private place by well-trained interviewers. Lastly, directions of causality could not be established due to the cross-sectional nature of the study. For instance, it remains unclear whether some factors, such as HIV education, contributed to recent HIV testing, or conversely, HIV testers were more likely to receive HIV education when they received HIV testing. Longitudinal studies are needed to address this shortcoming.

## Conclusions

Several factors associated with recent HIV testing among high-risk MSM in this study have been identified. The factors included exposure to HIV education, perception of high HIV risk, STI diagnosis, and condom use during sexual intercourse with both men and women and in anal intercourse with boyfriends. In Cambodia, significant efforts have been made to implement structural interventions with service packages for MSM. HIV education and social marketing should be expanded and specifically designed for MSM, addressing the increased risk of unprotected anal sexual intercourse and the importance of regular HIV testing for early enrolment in the care and treatment cascade.
